# Supplementary material for: Independent and interacting value systems for reward and information in the human brain
Source: eLife. 2022 Apr 13;11:e66358. doi: 10.7554/eLife.66358 (PMC9064296; doi:10.7554/eLife.66358)
Supplement: Supplementary file 1. — The table shows parameter estimates after fitting the model to participants’ data. Group mean and standard deviation are also reported for each parameter. [file elife-66358-supp1.docx]

Supplementary file 1. *Model estimated parameters from participants’ behavior.*

|  | **gkRL** | | | |
| --- | --- | --- | --- | --- |
| Participants | α | β | ω | Log(g) |
| Number1 | 0.004 | 7.916 | 0.457 | -1.895 |
| Number2 | 0.151 | 0.443 | 2.723 | 0.098 |
| Number3 | 0.684 | 0.189 | 17.52 | -13.81 |
| Number4 | 0.495 | 0.358 | 33.333 | -1.61 |
| Number5 | 0.518 | 0.201 | 12.980 | -1.46 |
| Number6 | 0.459 | 0.168 | 48.663 | -13.81 |
| Number7 | 0.528 | 0.147 | 9.181 | -0.748 |
| Number8 | 0.593 | 0.158 | 24.743 | -1.84 |
| Number9 | 0.658 | 0.214 | 33.661 | -1.84 |
| Number10 | 0.054 | 1.819 | 1.714 | 0.138 |
| Number11 | 0.761 | 0.155 | 31.631 | -18.07 |
| Number12 | 0.687 | 0.142 | 1.114 | -0.003 |
| Number13 | 0.660 | 0.125 | 29.913 | -2.64 |
| Number14 | 0.393 | 0.321 | 17.714 | -19.94 |
| Number15 | 0.23 | 0.338 | 4.808 | -0.134 |
| Number16 | 0.269 | 0.791 | 7.528 | -0.425 |
| Number17 | 0.046 | 0.244 | 3.032 | -2.43 |
| Number18 | 0.269 | 0.208 | 9.174 | -0.178 |
| Number19 | 0.011 | 1.562 | 0.508 | -0.642 |
| Number20 | 0.012 | 2.084 | 0.26 | -0.080 |
| Total | 0.374  (0.264) | 0.879  (0.176) | 14.53  (1.76) | 4.07  (6.51) |
