# Supplementary material for: Independent and interacting value systems for reward and information in the human brain
Source: eLife. 2022 Apr 13;11:e66358. doi: 10.7554/eLife.66358 (PMC9064296; doi:10.7554/eLife.66358)
Supplement: Supplementary file 2. — The table shows individual BIC for both standard RL and gkRL. [file elife-66358-supp2.docx]

Supplementary File 2. *Individual BICs .*

| **Individual BIC** | | |
| --- | --- | --- |
| **Subject** | **Standard RL** | **gkRL** |
| 1 | 263 | 166 |
| 2 | 224 | 167 |
| 3 | 188 | 150 |
| 4 | 271 | 83 |
| 5 | 195 | 153 |
| 6 | 280 | 110 |
| 7 | 209 | 184 |
| 8 | 244 | 153 |
| 9 | 267 | 109 |
| 10 | 263 | 114 |
| 11 | 243 | 145 |
| 12 | 173 | 181 |
| 13 | 253 | 165 |
| 14 | 210 | 127 |
| 15 | 229 | 166 |
| 16 | 223 | 92 |
| 17 | 279 | 280 |
