# Supplementary material for: Independent and interacting value systems for reward and information in the human brain
Source: eLife. 2022 Apr 13;11:e66358. doi: 10.7554/eLife.66358 (PMC9064296; doi:10.7554/eLife.66358)
Supplement: Supplementary file 3. — The table shows correlation coefficients between relative reward value and the covariates for each subject. [file elife-66358-supp3.docx]

Supplementary file 3. *Correlation of covariates with relative reward value.*

| **Subject** | **Max Value** | **Min Value** | **Standard Deviation** | **Averaged Value** | **Chosen-Second** |
| --- | --- | --- | --- | --- | --- |
| 1 | 0.135 | 0.135 | 0.718 | -0.123 | 0.938 |
| 2 | 0.333 | 0.333 | 0.657 | -0.013 | 0.889 |
| 3 | 0.115 | 0.115 | 0.672 | -0.116 | 0.917 |
| 4 | 0.504 | 0.504 | 0.574 | -0.061 | 0.884 |
| 5 | 0.162 | 0.162 | 0.649 | -0.282 | 0.924 |
| 6 | 0.441 | 0.441 | 0.586 | -0.048 | 0.892 |
| 7 | -0.082 | -0.082 | 0.65 | -0.461 | 0.938 |
| 8 | 0.539 | 0.539 | 0.54 | 0.054 | 0.881 |
| 9 | 0.355 | 0.355 | 0.488 | -0.049 | 0.882 |
| 10 | 0.34 | 0.34 | 0.649 | -0.163 | 0.927 |
| 11 | -0.025 | -0.025 | 0.603 | -0.319 | 0.920 |
| 12 | 0.386 | 0.386 | 0.551 | -0.105 | 0.920 |
| 13 | 0.646 | 0.646 | 0.547 | 0.074 | 0.881 |
| 14 | 0.327 | 0.327 | 0.584 | -0.13 | 0.914 |
| 15 | 0.511 | 0.511 | 0.646 | 0.03 | 0.890 |
| 16 | 0.396 | 0.396 | 0.638 | -0.016 | 0.896 |
| 17 | 0.474 | 0.474 | 0.709 | 0.018 | 0.897 |
| 18 | 0.327 | 0.327 | 0.714 | 0.101 | 0.918 |
| 19 | 0.101 | 0.101 | 0.681 | -0.247 | 0.923 |
| 20 | 0.117 | 0.117 | 0.68 | -0.14 | 0.941 |
| 21 | 0.143 | 0.143 | 0.595 | -0.062 | 0.900 |
