# Supplementary material for: Independent and interacting value systems for reward and information in the human brain
Source: eLife. 2022 Apr 13;11:e66358. doi: 10.7554/eLife.66358 (PMC9064296; doi:10.7554/eLife.66358)
Supplement: Supplementary file 4. — The table shows the 17 GLMs adopted in the fMRI data analysis all referring to activity associated with the onset of the first-free-choice trial. GLM0 and 5 are the univariate analyses, whereas the other GLMs relate with the model-based analysis. [file elife-66358-supp4.docx]

Supplementary file 4. *GLMs for fMRI data.*

| **NAME** | **REGRESSORS** |
| --- | --- |
| GLM0 | [Highest Reward choice; Lower Reward choice] |
| GLM0relexp | $[ {First free choice;RQ}_{t+1, j}\left( c \right);Q_{t+1, j}\left( c \right);24 motion regressors]$ |
| GLM0exprel | $[ {First free choice;Q_{t+1, j}\left( c \right);RQ}_{t+1, j}\left( c \right);24 motion regressors]$ |
| GLM0rew | $[ {First free choice;RQ}_{t+1, j}\left( c \right);Max Q_{t+1, j};Min Q_{t+1, j};mean Q_{t+1, j} ;24 motion regressors]$ |
| GLM1 | ${[First free choice;RQ}_{t+1, j}\left( c \right);24 motion regressors]$ |
| GLM1bis | ${[First free choice;Q}_{t+1, j}\left( c \right);24 motion regressors]$ |
| GLM2 | ${[First free choice;-I}_{t, j}\left( c \right);24 motion regressors]$ |
| GLM3 | ${[First free choice;-I}_{t, j}\left( c \right);{RQ}_{t+1, j}\left( c \right);24 motion regressors]$ |
| GLM3bis | ${[First free choice;{RI}_{t, j}\left( c \right);-I}_{t, j}\left( c \right);{{RT}_{t, j}; RQ}_{t+1, j}\left( c \right);24 motion regressors]$ |
| GLM4 | ${[ First free choice;RQ}_{t+1, j}\left( c \right);I_{t, j}\left( c \right);24 motion regressors]$ |
| GLM4bis | $[ First free choice; Q_{t+1, j}\left( c \right);{-I}_{t, j}\left( c \right); 24 motion regressors]$ |
| GLM4rew | ${[ First free choice;additional reward computations;RQ}_{t+1, j}\left( c \right);I_{t, j}\left( c \right);24 motion regressors]$ |
| GLM4diff | ${[ First free choice;aSD;RT;switch/stay;RQ}_{t+1, j}\left( c \right);I_{t, j}\left( c \right);24 motion regressors]$ |
| GLM5 | $[Defulat Option;NoDefault]$ |
| GLM6 | ${[ First free choice;Instrumental Info\left( c \right);-I}_{t, j}\left( c \right);24 motion regressors]$ |
| GLM7 | ${[ First free choice;-I}_{t, j}\left( c \right); Instrumental Info\left( c \right);24 motion regressors]$ |
| GLM8 | $[ First free choice; P(c/V_{t, j}\left( c_{i} \right));24 motion regressors]$ |
|  |  |
