# Supplementary material for: Independent and interacting value systems for reward and information in the human brain
Source: eLife. 2022 Apr 13;11:e66358. doi: 10.7554/eLife.66358 (PMC9064296; doi:10.7554/eLife.66358)
Supplement: Supplementary file 5. — The table shows brain activity not reported in the main text. PCC: Posterior Cingulate Cortex; mOFC: medial Orbitofrontal Cortex; aInsula: anterior Insula. [file elife-66358-supp5.docx]

Supplementary file 5 *Brain activity no reported in the text.*

| **GLM** | **ROI** | **Statistics** (cluster level correction) |
| --- | --- | --- |
| GLM0  *Highest reward - Lower Reward* | PCC (8, -44, 28)  PCC (-6, -22, 42)  mOFC (-10, 60, 16) | *p* < 0.001  *p* < 0.001  *p* = 0.037 |
| GLM1 (Beta > 0)  *Parametric Modulator: RelReward* | PCC (-8, -50, 28) | *p* < 0.001 |
| GLM2 (Beta > 0)  *Parametric Modulator: Information Gain* | right aInsula (34, 22, -8)  left aInsula (-34, 20, 6)  right dLPFC (42, 16, 40) | *p* < 0.01  *p* < 0.05  *p* = 0.001 |
| GLM2 (Beta < 0)  *Parametric Modulator: Information Gain* | PCC (-10, -34, 46) | *p* < 0.001 |
| GLM8 (Beta > 0)  *Parametric Modulator: Softmax probabilities* | PCC (-10, -32, 58) | *p* < 0.002 |
