# Supplementary figures and images for: Independent and interacting value systems for reward and information in the human brain
Source: eLife. 2022 Apr 13;11:e66358. doi: 10.7554/eLife.66358 (PMC9064296; doi:10.7554/eLife.66358)

Supplementary file 6 *Brain activity in GLM1bis.*


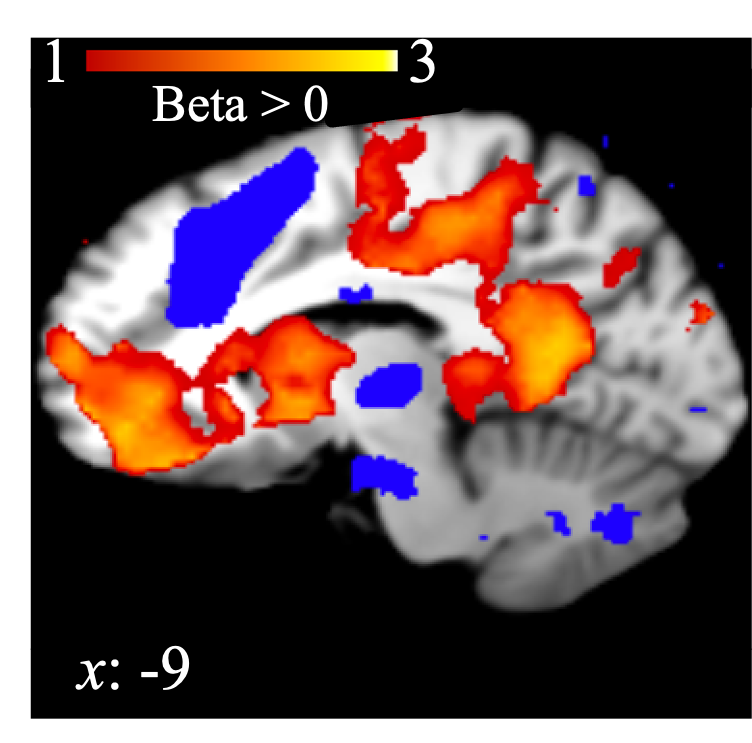

Supplement: Supplementary file 6. — The figure shows brain activity in GLM1bis [file elife-66358-supp6.docx]

Supplementary file 7 *Brain activity in GLM4bis.*


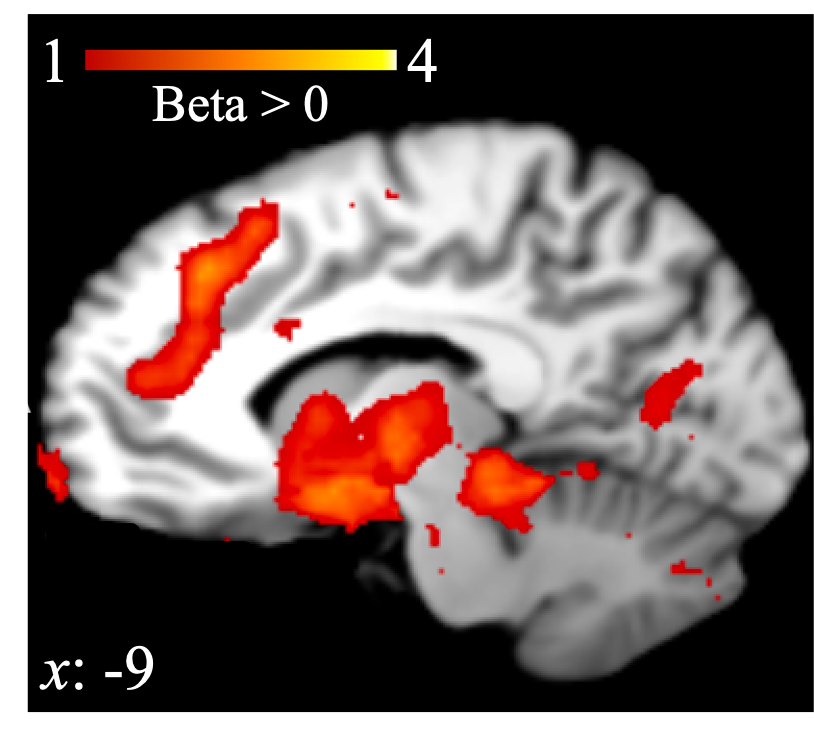

Supplement: Supplementary file 7. — The figure shows brain activity in GLM4bis [file elife-66358-supp7.docx]

Supplementary file 8 *Brain activity in GLM4rew.*


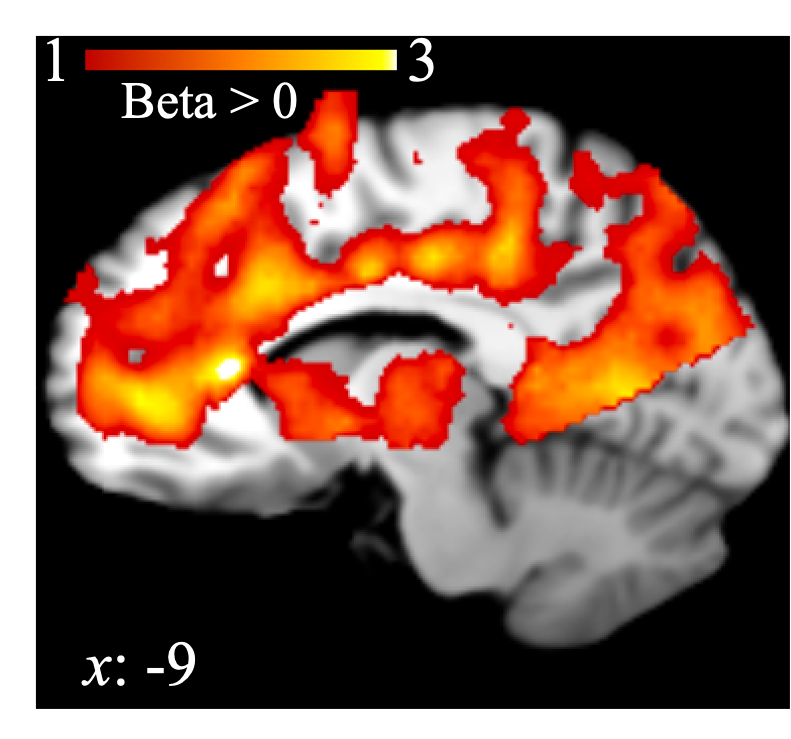

Supplement: Supplementary file 8. — The figure shows brain activity in GLM4rew. [file elife-66358-supp8.docx]

Supplementary file 9 *Brain activity in GLM3bis.*


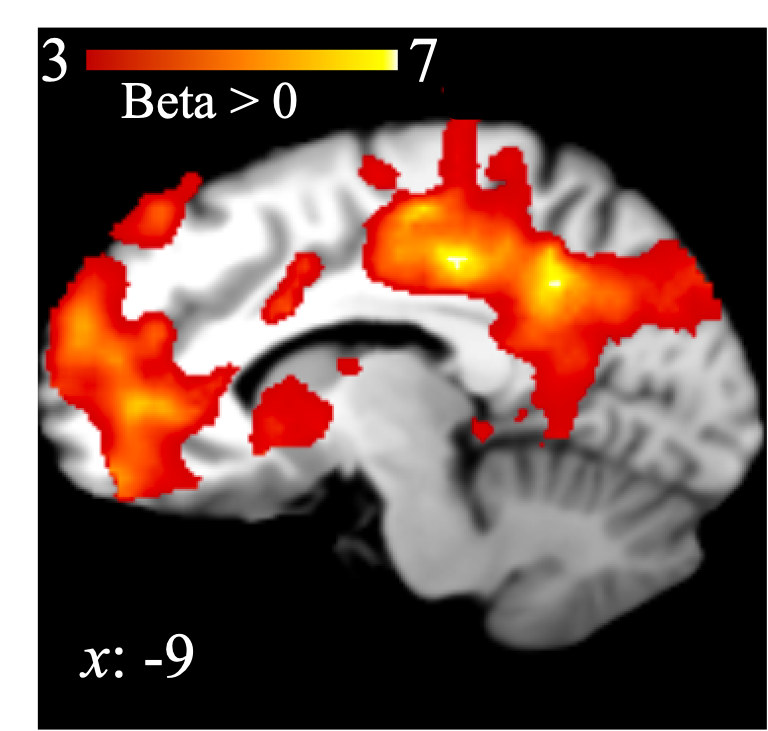

Supplement: Supplementary file 9. — The figure shows brain activity in GLM3bis [file elife-66358-supp9.docx]

Supplementary file 10 *Brain activity in GLM4diff.*


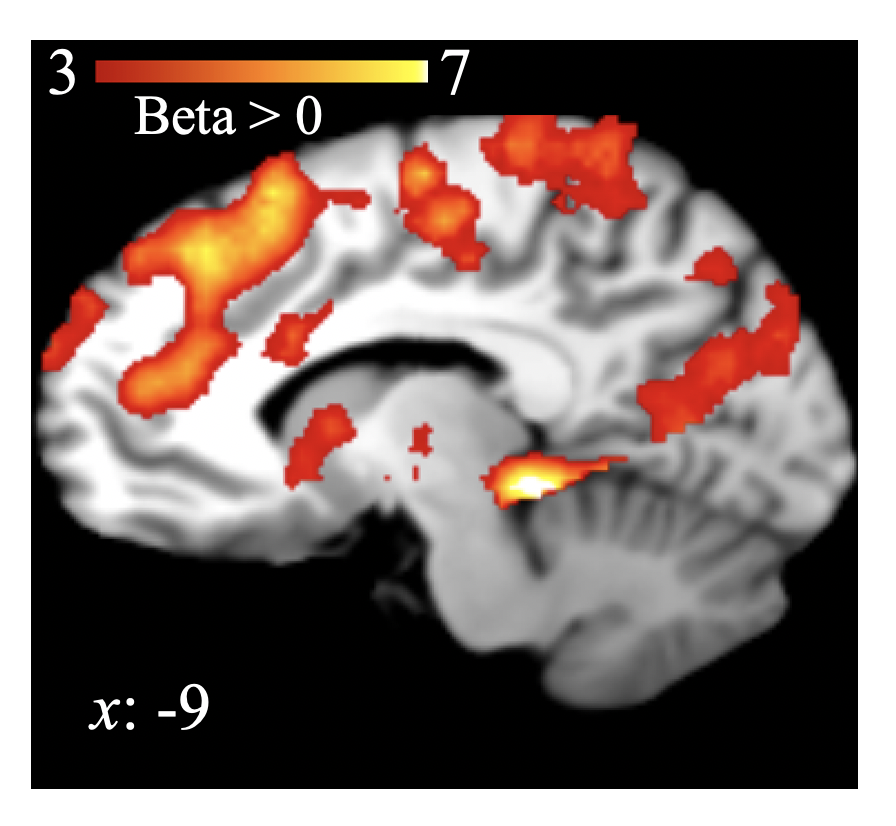

Supplement: Supplementary file 10. — The figure shows brain activity in GLM4diff [file elife-66358-supp10.docx]
