# Supplementary material for: Independent and interacting value systems for reward and information in the human brain
Source: eLife. 2022 Apr 13;11:e66358. doi: 10.7554/eLife.66358 (PMC9064296; doi:10.7554/eLife.66358)
Supplement: Supplementary file 11. [file elife-66358-supp11.docx]

Supplementary file 11 *Information Gain and Omega parameter.*

We re-ran our GLM2 analysis using the informationGain*omega regressor. The inclusion of a constant multiplier on a regressor has no effect on the significance of that regressor’s beta estimates. Significantly active voxels for our 1^st^ level analyses are identical whether the regressor is entered as only the InformationGain, or as InformationGain*Omega. However, because the regressor values were not standardized, the magnitude of the beta estimates depended on omega, i.e., the beta estimate of a voxel in the InformationGain*Omega analysis was simply the beta estimate obtained in the InformationGain analysis multiplied by omega. Again, this has no effect on the significance of the 1^st^ level analysis since changing the scale of a regressor does not change its relationship with the dependent variable (e.g., age is still significantly related to the passage of time regardless whether time is measured in years, days, or hours). However, the scale of betas from the parameter omega affects significance at 2^nd^ level.

Indeed, 2^nd^ level analysis of our data using the InformationGain*omega regressor (again, values were not standardized) produced no significant activity in dACC. This is not necessarily surprising, since omega reflects the degree to which subjective information is used to inform behavior in conjunction with reward value, and not the calculation of subjective information itself. Another way of saying this is that omega only has a meaningful interpretation in the context of a comparison with reward. Yet another way of saying this is that omega indirectly reflects reward value, and since our manuscript is premised on dissociating reward from information value, we would not necessarily expect to observe reward-related activity in a region associated with information value.

**To summarize:**

1. **the omega parameter in the gkrl model is not related to the calculation of subjective information value, but reflects the weighting of subjective information relative to reward value.**
2. **Regressing informationGain*omega against the BOLD signal does not influence significance at the 1^st^ level (see figure below).**
3. **At the 2^nd^ level, no activity in ACC is observed, but we never hypothesized that ACC activity reflects the comparison of reward to information which is implicitly coded by the omega parameter.**

**
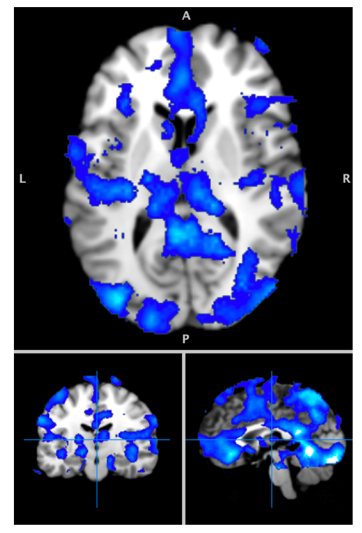
**

In the figure , we report 1^st^ level tmaps from Information Gain and Information Gain*omega from a randomly chosen subject. The figures shows only one color as activity for Information Gain and Information Gain*w perfectly overlap at 1^st^ level.
